# Heavy Silicone Oil and Intraocular Inflammation

**DOI:** 10.1155/2014/574825

**Published:** 2014-07-08

**Authors:** Francesco Morescalchi, Ciro Costagliola, Sarah Duse, Elena Gambicorti, Barbara Parolini, Barbara Arcidiacono, Mario R. Romano, Francesco Semeraro

**Affiliations:** ^1^Department of Medical and Surgical Specialties, Radiological Specialties and Public Health, Ophthalmology Clinic, University of Brescia, Viale Europa 15, 25123 Brescia, Italy; ^2^Department of Health Science, Ophthalmology Clinic, University of Molise, Via De Sanctis 1, 86100 Campobasso, Italy; ^3^Dipartimento di Oftalmologia, Istituto Clinico Sant'Anna, Via del Franzone 31, 25126 Brescia, Italy; ^4^Department of Neurological Sciences, Reproductive Sciences and Dentistry, Federico II University, Via Pansini 5, 80121 Naples, Italy

## Abstract

In the past two decades, many advances have been made in vitrectomy instrumentation, surgical techniques, and the use of different tamponade agents. These agents serve close retinal breaks, confine eventual retinal redetachment, and prevent proliferative vitreoretinopathy (PVR). Long-acting gases and silicone oil are effective internal tamponade agents; however, because their specific gravity is lower than that of the vitreous fluid, they may provide adequate support for the superior retina but lack efficacy for the inferior retina, especially when the fill is subtotal. Thus, a specific role may exist for an internal tamponade agent with a higher specific gravity, such as heavy silicone oils (HSOs), Densiron 68, Oxane HD, HWS 45-300, HWS 46-3000, and HeavySil. Some clinical evidence seems to presume that heavy tamponades are more prone to intraocular inflammation than standard silicone if they remain in the eye for several months. In this review, we discuss the fundamental clinical and biochemical/molecular mechanisms involved in the inflammatory response after the use of heavy tamponade: toxicity due to impurities or instability of the agent, direct toxicity and immunogenicity, oil emulsification, and mechanical injury due to gravity. The physical and chemical properties of various HSOs and their efficacy and safety profiles are also described.

## 1. Introduction

The introduction of silicone oil (polydimethylsiloxane, PDMS) to retinal detachment surgery in the early 1980s was one of the main steps in the effective treatment of this pathology [1–4]. In the last three decades, vitreoretinal surgery combined with PDMS tamponade has become the widespread treatment for complicated cases of retinal detachment caused by a proliferative process. Silicone application mainly serves two functions. The first is the displacement of the retina toward the eye-wall by its surface tension effect and volume displacement, and the second, to a lesser degree, is the tamponade of the superior retina by its flotation force.

More than 30 years of clinical use has demonstrated that the tamponade effect of PDMS is usually sufficient, provided that the retina is completely mobile and provided that no new membranes develop. Moreover, the stability and immunological tolerability of PDMS make it relatively safe as a long-term internal tamponade. Histological examination of the human retina after more than 3 years of PDMS endotamponade did not show significant morphological alterations [5]. Intraretinal or intracellular deposits suggestive of silicone have been observed in attached retinas only if subretinal silicone deposition occurred in accidental situations [5].

However, PDMS and long-acting gases provide good support only for the superior retina and lack efficacy for the inferior retina, especially when the fill is subtotal. This makes these tamponade agents less useful for closing inferior retinal breaks and for defending them from the proliferative vitreoretinopathy (PVR) that usually begins in the inferior quadrants. Placing an agent that is heavier than water in contact with the retina should reduce the redetachment rate and the rate of inferior PVR.

In the past two decades, clinicians and researchers have attempted to identify internal tamponades that are heavier than water and have good tolerability. The first heavy tamponade used was fluorinated silicone oil or fluorosilicone (FSiO), but its high rate of complications such as early emulsification and development of intraocular inflammation and PVR limited its use [6]. A second group of heavy internal tamponades, the perfluorocarbon liquids (PFCLs), was studied for prolonged postoperative endotamponade at the end of the 1980s. These are fully fluorinated alkane compounds with a high specific gravity. However, these compounds turned out to be unsuitable long-term internal tamponade because of the mechanical damage on the retina and the tendency for droplet dispersion [7–9]. Presently, these compounds are widely used as intraoperative tools, but not as vitreous substitutes.

A third group of substances, liquid semifluorinated alkanes (SFAs), appeared to have the potential to act as heavy internal tamponade agents [10]; in particular, perfluorohexyloctane (F6H8) seemed to be well tolerated in long-term animal studies [11]. In clinical practice, the use of F6H8 provided adequate reattachment rates and few signs of retinal damage; however, it was associated with a high rate of postsurgical inflammation and an early rate of emulsification of droplets into the entire eye [12].

SFAs have the ability to bring the silicone oil into solution, creating a fourth category of heavy tamponades, the heavy silicone oils (HSOs). HSOs are admixtures of different concentrations of highly viscous PDMS and SFAs, combining the advantages of increased gravity and high viscosity. Some of these mixtures were more tolerated by ocular tissues compared to SFAs, and these mixtures have been successfully investigated as long-term endotamponades. However, in some clinical situations, the combination of two or more tamponade agents is suspected to increase postsurgical inflammation.

Two compounds belonging to the HSO group are available for clinical use: Densiron 68 (Fluoron: a combination of F8H8 and silicone oil) and Oxane HD (Bausch and Lomb: a combination of olefins RMN3 and silicone oil). A third compound, HWS 46-3000, appeared to be well tolerated, but it is not yet available in common practice.

In this review, we describe the current knowledge on HSOs and heavy tamponades and discuss the fundamental clinical and biochemical/molecular events involved in the ocular inflammation induced by these compounds.

### 1.1. Physical Properties of an Optimal Heavy Tamponade

The essential attribute of the PDMS is its ability to keep the retina in contact with the pigment epithelium by the hydraulic force of its volume displacement, thereby alleviating the tractions. The efficacy of an internal tamponade depends on its ability to make contact with the internal surface of the retina.

The PDMS cannot flatten the retina because it has a weak flotation force; rather, it exerts a tamponade effect mainly by immobilizing the retina and reducing fluid circulation. Thus, the tamponade effect of the injected PDMS is modest and is not comparable to that of air or gas. A layer of fluid between the retina and the silicone bubble is always present, and closed contact between the oil and retina is not possible. However, the stabilization of the eye for a long period after surgery is the main advantage of silicone oil compared to gas.

PVR is an exaggerated wound-healing phenomenon in which inflammation, proliferation, and remodeling lead to a retinal scar [13]. At the end of surgery, the meniscus of fluid that remains between the endotamponade and the retina is a milieu of rich proinflammatory cytokines and growth factors that promote PVR development, and this is the main cause of failure after retinal detachment surgery.

With conventional “light” endotamponades (either gas or PDMS), the PVR is located in the inferior quadrants where the remnant fluid is displaced in almost all cases. In cases of inferior breaks, the contact between an agent that is heavier than water and the inferior retina can prevent the passage of aqueous through the hole and displace water upwards. An ideal heavy tamponade agent should possess the following qualities: optical clarity, no effects on the eye's refractive state, no toxic effects on eye structures, no effects on eye pressure, no cataractogenic effects, and the ability to inhibit inflammation, cellular migration, and glial proliferation [14]. Moreover, the following physical properties regulate endotamponade effectiveness: the difference in the specific gravity of the agent and the aqueous (buoyancy), the interfacial tension, and the viscosity [15]. Unfortunately, all of the presently used agents have both advantages and disadvantages related to their different properties.

The specific gravity (the difference between the specific gravity of the agent and water) determines whether the tamponade will sink or float in water and the shape of the intraocular bubble. The specific gravity and the interfacial tension determine the effectiveness of an internal tamponade in the short term. The viscosity of the material is crucial for maintaining its integrity, thus reducing dispersion in the long term. In contrast to PDMS (specific gravity, 0.97 g/cm^3^), the high specific gravities of perfluorodecalin (1.93 g/cm^3^) and F6H8 (1.35 g/cm^3^) allow these substances to stay perfectly in contact with the lower retina. These compounds are able to flatten the retina because of their strong sinking force; they fit perfectly over all of the irregularities of the posterior pole and the recesses of the indents, and no fluid remains between the inferior retina and the tamponade agent. However, the specific gravity of these agents is probably too high, and the absence of water between the agent and the inferior retina produces a mechanical or metabolic negative effect that impairs retinal function [16]. The lower specific gravities of “lighter” heavy tamponades, such as Oxane HD (specific gravity, 1.02 g/cm^3^) and Densiron 68 (1.06 g/cm^3^) minimize these effects. Thus, these compounds should be less toxic, although this reduces their tamponade effects, especially in the presence of a retinal indents [17].

Further, an effective tamponade must have a high interfacial tension against water in order to push the retina toward the eye-wall. Gas or air has the highest interfacial tension against water (approximately 80 mN/m), whereas PFCLs and silicone oil derivatives (PDMS or HSO) have a lower tamponade capability because of their lower interfacial tension against water (around 40–45 mN/m or 35 mN/m, resp.).

According to Archimedes' principles, the tamponade force that presses against the retina depends on the gravity of a submerged bubble, namely, buoyancy. When in contact with water, the bubble of a light or heavy silicone oil is rounded because of its small “pressing” force. In an eye that is almost completely filled with a tamponade, this substance is in contact with only a portion of the retina (superior or inferior, depending on the gravity), while it forms a convex meniscus on the opposite side that is not in contact with the retina. The shape of this meniscus is more or less convex, depending on the physical characteristics (gravity, buoyancy, and superficial tension) of the substance; in general, a flat meniscus is a characteristic of a good endotamponade agent. For example, gas or air has a flat meniscus, while PDMS and HSO have a convex meniscus, and it seems that the Densiron 68 meniscus is less convex than the Oxane HD meniscus.

In clinical practice, it remains unclear whether the differences between Densiron 68 and Oxane HD are significant. The essential role of any vitreous substitute is presumably its ability to fill the eye and maintain the retina in contact with the pigment epithelium rather than to flatten it [18].

Emulsification is a frequent complication associated with the use of heavy internal tamponade [19]. This phenomenon is influenced by many factors including the interfacial tension, the viscosity of the oil, and the presence of impurities such as low-molecular-weight siloxanes and catalytic remnants [15].

The viscosity rate is the main factor influencing emulsification; a reduction in viscosity reduces the mechanical energy needed to disperse a large bubble in small droplets. In theory, an intraocular tamponade should be highly viscous, thus reducing the tendency to emulsify and to disperse into small bubbles that can cross retinal breaks or the zonula to the anterior segment, causing inflammation or glaucoma [20]. Silicone oil, which has high viscosity (5000 mPas), is more stable and tends to have less dispersion; therefore, it is associated with a lower rate of complications related to emulsification compared to the less viscous PDMS (1000 mPas) [21].

In the clinical practice, however, the PDMS is usually removed after 3-4 months, and such a difference in dispersion may be not significant in this time interval. The high viscosity of 5000 mPas PDMS increases the difficulties associated with handling the substance. A PDMS of 1000 Cs can be introduced and removed much more easily than a PDMS of 5000 Cs; thus, the former is largely utilized by most vitreoretinal ophthalmologists. Moreover, with the advent of minimally invasive surgery (23–25 gauge), the use of a less viscous silicone oil is preferable in order to save time during its introduction and its passage through the small gauge system. Therefore, obtaining an HSO of low viscosity that does not generate the phenomena of emulsification would be desirable.

Heavy tamponades have lower viscosity than PDMS: F6H8 and the other SFAs have a viscosity of 2.5–3 mPas, close to that of water (1 mPas). These compounds are easy to handle, but they tend to emulsify very early after surgery. Dispersion was described in 30% to 100% of cases treated after a few weeks with F6H8, depending on the time to removal. The mixture of SFA with a PDMS that has a viscosity of 1000 mPas can inhibit dispersion by F6H8, but this mixture was found to be unstable, depending on the temperature and movement of the eyes [22, 23].

The mixtures of an SFA and a PDMS with a viscosity greater than 5000 mPas, forming the HSO compounds, seem to be more stable. HSO compounds have higher viscosity than pure FSA: approximately 1400 mPas for Densiron 68 and approximately 3800 mPas for Oxane HD. Although this quality slows the emulsification rate, it influences their handiness during removal [24].

The amount of emulsification of heavy tamponades is, among other factors, time-dependent. Thus, the tendency to emulsify is the main factor that influences the time to removal of these tamponades.

This factor is crucial for stabilizing the retina for the time that is necessary for PVR to develop (usually 4–6 weeks). The better tolerance of the new HSOs allows these substances to remain for up to 3-4 months without detrimental effects [25].

### 1.2. Immune Response and the Proinflammatory Nature of HSOs

The inflammatory response after prolonged retinal detachment and after vitreoretinal surgery peaks in the development of PVR, which occurs when the retinal cells are exposed to the inflammatory milieu in the vitreous humor [26]. The “PVR soup” consists of the aqueous humor containing growth factors and cytokines [27]; it tends to settle at the level of the inferior retina and posterior pole because of gravity [28]. This situation is common in complicated retinal detachment, but it is amplified after invasive surgery and by the use of intraocular tamponades that float over a subtle film of liquid where the inflammatory cytokines and growth factors reach the critical concentration over the inferior retina.

The accumulation of the PVR soup beneath the inferior meniscus of the PDMS or gas exposes the inferior retina (in the orthostatic position) and the posterior pole (in the supine position) to factors that may generate epiretinal membranes. Heavy tamponades theoretically possess the quality to displace this inflammatory environment away from the inferior retina and the posterior pole [29]. With a heavy tamponade, the head movements during common daily postures should frequently displace the liquid meniscus from the upper retina to the posterior capsule of the lens. In contrast, with PDMS, head movements frequently displace the liquid from the inferior retina to the posterior pole, increasing the risk of damaging the macula. However, PDMS has been used for more than three decades and is appreciated for its stability and immunological tolerability, which make it safe for use as a long-term internal tamponade. The same level of safety has not yet been achieved by any of the heavy tamponades used up to now, especially for their physical and immunological interaction with ocular tissues.

Many authors have noted that heavy tamponades are more prone to causing intraocular inflammation compared to standard silicone if they remain for several months in the eye. It is difficult and often impossible to distinguish between inflammation caused by the tamponade and the inflammatory reaction that is associated with the underlying complicated retinal disease. High inflammation can be commonly expected after a complicated retinal detachment surgery, and this is not related to the tamponade used. Fibrin formation, corneal edema, and cataract progression are frequent complications related to surgical trauma or to the ocular disease itself (i.e., in cases of retinal detachment after an ocular injury). Moreover, severe reproliferation is the major reason for anatomical and functional failure, and it can be seen with or without the use of heavy tamponades.

However, detecting any possible adverse inflammatory event related to the physical characteristics of any endotamponade agent is crucial because it could modify or amplify the wound-healing response and stimulate PVR, which is the primary reason for visual loss and poor visual outcome.

Four mechanisms are involved in the genesis of the inflammatory response: toxicity due to impurities or the instability of the agent, direct toxicity and immunogenicity, oil emulsification, and mechanical injury due to gravity [30].

### 1.3. Toxicity due to Impurities or the Instability of the Agents

PDMS and FSiO contain impurities like linear and cyclic low-molecular-weight components (LMWCs), ionic compounds, and compounds with cleavable fluoride that are thought to cause ocular toxicity [31]. LMWCs (less than 2,500 Da) have high volatility and may diffuse as vaporized molecules into the surrounding tissues, where they can produce toxic effects. The vaporized siloxanes can also condense and become silicone oil droplets in areas of temperature change, such as near the iris or in the anterior chamber, or in presence of polarized molecules in the anterior chamber fluid. Further, the inactivated catalysts remaining in the silicone oil may be toxic.

Severe inflammation and corneal edema can be induced when small species of linear and cyclic LMWCs of endotamponades are injected into the anterior chambers of animals. The ocular responses to the single species of the LMWCs increase as the molecular weights decrease. However, unpurified PDMS and FSiO, as well as purified oils (via solvent fractionation), usually do not cause significant adverse ocular responses, presumably because the amounts of LMWCs (especially the smallest species) in the oils are relatively small.

Using gas chromatography, several authors analyzed the PDMS and FSiO recovered from rabbits and human vitreous cavities up to 2 years after injection and discovered that LMWCs may diffuse from the oils into the ocular tissues [32]. Although the long-term effect of LMWCs in the intraocular PDMS and FSiO has not been determined, the diffusion of LMWCs into ocular tissues may be related to the chronic ocular toxicity of the oils. In addition, postoperative emulsification of silicone is related to the number of low-molecular-weight polymer chains [32].

In HSOs, the semifluorinated alkanes are embedded in silicone oil molecules that may theoretically contain LMWCs. However, the companies that produce Densiron 68 and Oxane HD stated that these agents are 100% pure preparations and that they do not contain low-molecular siloxanes and other impurities.

The biocompatibility of the SFAs and their admixtures with PDMS (the HSO) depends on the lipophilic behavior and on the molecular dimension of the semifluorinated alkanes. Because cell membranes and other physiological borders are composed of lipophilic substances, it is possible that they could be damaged or solubilized into the silicone bubble at certain temperatures [10–12]. The composition of the HSO may vary with time and temperature and from contact with other chemical agents. For example, the higher temperature of the anterior chamber might separate the F6H8 and PDMS in some situations.

Further, the stability of the combination of two different agents may cause unexpected ocular toxicity. The interaction between F6H8 and other substances like PFCLs, PDMS remnants, or the cortical humor vitreous and humor acqueous may alter the stability between the two compounds and the properties of HSOs. The decomposition of these substances was shown to cause intraocular inflammation or phenomena like “sticky silicone oil” [33, 34]. It was shown that F6H8 might react with remnants of the humor vitreous and humor acqueous either in the vitreous base or in the posterior pole, creating whitish epiretinal membranes [35].

Even if an apparently complete exchange of the PFCL with air is assumed, a thin layer of the PFCL may remain on the retinal surface and in ciliary bodies; these remnants can be found in droplets in many patients months or years after the surgery at the follow-up visits [36].

The interaction between the HSO and the volatile remnants of the PFCL or vitreous remnants may generate drops of sticky silicone, a sort of “glued oil,” attached on the retinal surface and, in the worst cases, on the macula. PFCL remnants were found in high concentrations in the sticky samples of several patients [33]. Contamination of the tamponade with the heavy liquids used during intraoperative manipulations is also suspected to cause granulomatous uveitis with the use of Oxane HD [37]. For these reasons, it is recommended that a PFCL-air exchange be performed before injecting any HSO in order to avoid direct contact between the PFCL and HSO, thus preventing unpredictable side effects.

The biocompatibility of the SFA and their admixtures with PDMS (the HSO) is dependent on the lipophilic behavior and on the molecular dimension of the semifluorinated alkanes. Because cell membranes and other physiological borders are composed of lipophilic substances, it is possible that they could be damaged or solubilized into the silicone bubble at certain temperatures.

### 1.4. Direct Immunogenicity and Toxicity of the Compounds

The early clinical reports of some heavy tamponades showed a relatively high rate of intraocular inflammation. A fibrinoid reaction and even retinal necrosis associated with the use of high-density fluorosilicone oils as well as semifluorinated alkanes such as F6H8 and their oligomers have been reported [12, 35, 38–40].

In particular, F6H8 is suspected to increase the wound-healing reaction and to cause granulomatous reactions, fibrinoid reactions, and retropupillary membrane formation. The direct immunogenicity of this compound has been demonstrated by the finding of a granulomatous reaction with epithelioid cells containing minute drops of F6H8 [37].

The introduction of HSO reduced the rate of intraocular inflammation compared to previous reports. However, several cases of fibrin formation and unusual anterior chamber inflammation were reported either with Oxane HD or with Densiron 68 [37, 41]. An abnormal inflammatory reaction was not found in any patients treated with HWS 46-3000 [42].

The chronic presence of an intraocular endotamponade may also indirectly cause some form of toxicity. An endotamponade that remains in the vitreous cavity for several months may absorb endogenous substances from the ocular tissues or exogenous substances via the blood stream. The analysis of PDMS and FSiO extracted after several months of intraocular placement demonstrated the presence of cholesterol, retinol, and lipophilic acids that were extracted from the retinal cells or from the blood. Further, depending on their molecular dimensions and temperatures, SFAs may extract cholesterol from ocular plasma membranes that are damaged or are solubilized into the silicone bubble [10]. These findings suggest that intravitreal endotamponades containing PDMS or SFAs are not completely inert and may extract cellular components or accumulate substances not normally present in the vitreous cavity, and these substances may have a cytotoxic effect over time [43, 44].

### 1.5. Emulsification

Heavy tamponades with a viscosity that is lower than that of silicone oil are more prone to emulsification compared to standard silicone oil, which in turn gives rise to inflammation. The dispersion and diffusion of a tamponade agent in the aqueous are responsible for the subsequent formation of an emulsion of droplets or “fish eggs” [45]. Emulsification is probably either the effect or the cause of intraocular inflammation, quite apart from the fact that individual agents might be a stimulant for inflammatory reaction. Intraocular inflammation promotes early emulsification of the endotamponade, while the diffusion of foreign molecules from the endotamponade promotes further inflammation.

Minute bubbles of oil are suspected to trigger inflammatory cell chemotaxis and phagocytosis, which stimulate a foreign body-type reaction [46]. However, it is not clear whether the size of the bubble or the combination of the vesicle shape with a specific stabilizing surfactant activates neutrophils or stimulates phagocytosis by monocytes [47].

Dispersion also depends on the underfilling of the tamponade after surgery especially in large-volume eyes and if severe postoperative inflammation coexists. Silicone oils are composed of polymers and hence show the characteristics of non-Newtonian fluids, which means that the viscosity changes along with the share rate. Saccadic and pursuit movements of the eyes and of the head may cause intraocular fluid currents that exert shear stress on the silicone bubble surface. Therefore, the shear force or the lateral attrition, created by rotatory movements, exceeds the surface tension of the bubble, creating a dispersion of small fractions of the tamponade in small bubbles.

Because the viscosity of silicone oil is determined by its molecular weight, low viscosity silicone emulsifies more easily. Differences in the rates of emulsification are not due to differences in surface tension because surface tension changes minimally with increasing viscosity. Different samples of silicone oil with the same viscosity may be composed of a narrow band of different molecular weight chains containing only a few short chains, whereas another sample of the same viscosity may be composed of a wider range of molecular weight chains with more short-chain molecules capable of emulsification. The homogeneity of the silicone components and the low concentration of the LMWCs are important factors for avoiding toxicity and emulsification.

While emulsification is transitory in the first phase, it becomes permanent in the presence of blood components and inflammatory proteins that act as surfactants [47]. Red blood cell membranes, plasma lipoproteins, and HDL-apolipoproteins support silicone oil emulsification [48]. Further, vigorous physical activity with the tamponade in situ is reported as a possible cause of dispersion, opacification of the endotamponade, and intraocular inflammation [35]. Finally, the contact of silicone oil with any type of substance during a direct exchange may increase emulsification [49].

The first agents used as heavy tamponades (FSi, PFCL, and F6H8) have low viscosity and fast intraocular emulsification; however, the resistance to extensional deformation and therefore the extensional viscosity of F6H8 may be increased by mixing a certain amount of very long-chain silicone molecules into the heavy tamponade. This maintains the specific weight at a value greater than 1 and increases the resistance to emulsification.

Rachel et al. studied a combination of high-molecular-weight (423 kDa) PDMS and silicone oil 1000 at 5% and 10% w/w concentrations in order to increase the emulsification resistance of the tamponade agents while maintaining ease of injection and removal [50].

HSOs are derived from a mixture of a highly viscous PDMS (more than 5000 mPas) and different semifluorinated alkanes (F6H8, F4H5, and F4H6) or a similar substance (RMN-3), and these have a lower tendency to create dispersion and emulsion. However, the concentration of the two components may vary with time and temperature, and the possible chemical decomposition of HSO has been reported, where the heavier component tends to settle over time in the inferior part of the bubble, separating it from PDMS. Thus, the specific gravity of HSO in the eye may become heterogeneous over time with the oil because the SFA dissociates from the silicone oil, thereby producing droplets of PDMS and droplets of SFA. This dissociation may result in an anterior uveal reaction [51, 52]. The iris pigment could be absorbed by HSO droplets in some cases, leading to iris depigmentation [52].

In an in vitro model, Caramoy et al. demonstrated that increasing the extensional viscosity by the addition of small amounts of very long-chain silicone molecules significantly influenced the reduction of the emulsification for 1000 cSt silicone oil (Siluron 2000) and for 1000 cSt silicone oil with an admixture of F6H8 (Densiron 68 HV) [53]. These findings are expected to be investigated further in an in vivo model.

### 1.6. Effect of Gravity in Long-Term Vitreous Tamponade

Previous reports showed that PFCL agents (perfluorodecalin, perfluoroperhydrophenanthrene, and perfluorooctane) are clinically tolerated in the eyes for only a few days (5–7 days) [54–57]. Mechanical pressure on the retina may be partly responsible for the changes observed in the retina when PFCL agents are used. These considerations are mainly dependent on experiments and histological evaluations conducted in animal models. A few weeks of endotamponade with PFCL may cause the following ultrastructural changes in the inferior retina of rabbits: narrowing of the outer plexiform layer, ultrastructural distortions of the photoreceptor outer segments, and migration of the receptor cell nuclei to the photoreceptor layer [8, 9, 58]. These changes may represent a mechanical rather than toxic effect; in fact, similar changes have been reported in the superior retina in silicone-filled eyes. The specific gravity of PFCL ranges from between 1.7 g/cm^3^ and more than 2.0 g/cm^3^. The histologic changes in the retina may be partly attributed to the dystrophic effect of the “heavy” liquids that press the inferior retina. However, it was noted that the retinal damage was more evident in the external layers rather than in the inner retinal layers that are in direct contact with the heavy substance. A mechanism of damage different from a simple mechanical interaction was assumed.

Recent observations indicate that PFCL toxicity is not primarily due to the high specific gravity or possible chemical impurities but rather due to their inability to dissolve ions. Gravity might not be causally linked to retinal damage that may rather depend on a metabolic disturbance. OCT measurements indicate that PFCLs, including the semifluorocarbon PFH with low specific gravity, replace most of the aqueous sink volume available for potassium (K^+^) siphoning. Thus, impairment of retinal K^+^ clearance may be an important mechanism of PFCL-induced retinal injury.

These observations explained the morphological alteration reported regarding Müller cells. Müller cells have been shown to develop features of reactive gliosis including hypertrophy, expression of glial fibrillary acidic protein, and drop-like protrusions between the inner segments of the photoreceptors. Müller cells may be directly injured by the elevated [K^+^], thus causing subsequent atrophy of the photoreceptors that occupy the external retinal layers. HSO is less efficient compared to PFCLs and SFAs at remaining in contact with the retina and is unable to fit into small recesses; however, this relatively poor contact allows a thin film of aqueous to remain in contact with the retinal surface, and this is important for retinal cell survival and for potassium siphoning by retinal Müller cells [16].

## 2. Internal Tamponade Agents

### 2.1. Fluorosilicone

Fluorinated silicone oil (trifluoropropylmethylsiloxane or fluorosilicone-FsiO), which has a density of 1.30 g/cm^3^, was the first heavy tamponade used. Clinically, it was marked by immediate, albeit transient, iritis. The ocular toxicity of fluorinated silicone oils was attributed to their low-molecular-weight components and to their high dispersion rate [31, 32]. In animal models, FsiO fluorosilicone caused inflammatory responses that exceeded those observed with PDMS [6]. This agent is thought to promote PVR in the longer term, with an epiretinal membrane forming around the oil bubble. Histologically, these membranes showed foreign body reactions [59].

A copolymer of PDMS and FsiO was evaluated in order to avoid the anatomical damages caused by PFCLs with the aim of decreasing the specific gravity (density, 1.16 g/cm^3^) of the tamponade [60].

The atrophic retinal changes were much less than those observed with the heavier perfluorotetradecahydrophenanthrene (density, 2.03 g/cm^3^). However, thinning of the outer plexiform layer in rabbit retina was still observed after 6–8 weeks and small droplets ingested by mononuclear cells were found in the vitreous cavity or preretina after 4–6 months [61, 62].

### 2.2. Perfluorohexyloctane (F6H8)

Perfluorohexyloctane (F6H8) is the most extensively investigated agent belonging to a group of fluorinated hydrocarbons: the semifluorinated alkanes (SFA) [10]. These agents have specific gravities greater than those of water, but slightly lower than those of perfluorooctane (1.35 g/cm^3^), and their surface tension and interfacial tension against water are equal to those of perfluorocarbon liquids (45.3 mN/m).

F6H8 is chemically and physically inert because of the strength of its hydrocarbon (C–H) and fluorocarbon (C–F) bonds. The fluorocarbon moiety is lipophobic, while the hydrocarbon moiety is lipophilic; thus, the SFAs are amphiphilic molecules that are soluble in both silicone oils and perfluorocarbon liquids but are insoluble in water.

F6H8 is a biocompatible compound that was investigated as a candidate for blood substitutes [63]. F6H8 was well tolerated for three months in rabbit eyes [11], and it was introduced initially as a solvent for silicone oil to remove silicone oil remnants from intraocular surfaces [64, 65]. Further, F6H8 was investigated as an intraoperative tool and as a long-term tamponade in several small case series [12, 66]. Its low density and viscosity (2.5 mPas) reduced the risk of mechanical retinal damage, but it promoted dispersion and the phenomena of emulsification in the eye in up to 100% of all treated cases [67].

According to some authors, the ability of this compound to generate inflammatory responses is mostly due to its propensity to disperse and to form small, emulsified droplets. Minute bubbles of oil subsequently trigger chemotaxis of inflammatory cells and phagocytosis [46, 47]. Despite good results with the use of F6H8 in animal models and in some small case series [11], studies conducted in vitro and in vivo showed evidence that F6H8 had proinflammatory activity. In preclinical studies, blood-retinal barrier breakdown associated with local vasoconstriction, hypertrophy of Müller cells, and vacuolization of the inner retinal surface were observed in rabbit retinas after 6 weeks of tamponade [68].

An evaluation using the live/dead assay on cultured ocular cells that were incubated with F6H8 for up to 5 days showed a significant reduction of vital EPR cells. Due to its lipophilicity, F6H8 seemed to be able to interact with cell membranes, causing a change in the adherence of the cells to extracellular matrix [69].

Some evidence for an irritating effect has been observed in clinical pilot studies using F6H8 as a retinal tamponade. In some cases, retrolental, epiretinal, and simil-PVR membranes were associated with its use as a prolonged tamponade. These membranes were similar to the classical PVR membrane histologically, but they also exhibited dense macrophagic infiltration and foreign body reactions. Further, they contained vacuolated and pigmented CD68-positive cells, exhibiting a macrophagic and EPR phenotype. These observations supported possible differentiation of the EPR cells in response to the proinflammatory stimulus induced by F6H8 [35].

The presence of intracellular droplets of F6H8 in the vacuolated cells suggested that the contact with the oil in the form of microemulsion causes activation of the monocyte-macrophage population. This finding indicated that the inflammatory reaction was enhanced by droplets of a certain vesicle size. In an in vitro study, however, the inflammatory response appeared only when the vesicles interacted with specific artificial, but not natural surfactants [46].

From the clinic-pathological point of view, the inflammatory reaction leads to the formation of epiretinal membranes that sometimes extend to the posterior surface of the lens. The difference between these membranes and the classical ones encountered in PVR is greater infiltration of leukocytes, which appear to be mostly CD68-macrophages, or rather RPE cells, which have “transdifferentiated” to a macrophage-like phenotype [47]. Epithelioid cells, which are typical of a granulomatous reaction, were found in some specimens, suggesting that emulsified F6H8 could result in the release of growth-promoting factors for macrophages.

Regarding the development of retrolental membranes, it is known that silicone oil usually causes cataract formation because it interferes with the metabolism of posterior capsule epithelial cells [70].

The microscopic examination of lens capsule in eyes after F6H8 tamponade demonstrated the presence of macrophages adhering to the lens capsule with epithelioid cells and with fibroblastic differentiation, thus adding a probable inflammatory genesis to cataract formation [47].

### 2.3. Other Perfluoroalkanes Oligomers: Perfluorobutylpentane (F4H5), Perfluorobutylhexane (F4H6), and Perfluorobutyloctane (F4H8)

A recent study by Mackiewicz et al. conducted on rabbits showed that the use of different semifluorinated alkanes leads to quite different immunologic reactions. Whereas F6H8 (perfluoroexyloctane) and F4H5 (perfluorobutylpentane) were well tolerated, F4H6 (perfluorobutylhexane) and F4H8 (perfluorobutyloctane) resulted in a severe inflammatory response, which appeared to be more pronounced when these substances were used in pure form rather than in an admixture with silicone oil. Microscopic investigation showed that the vitreous was replete with immune cells, mostly neutrophils.

Chemically, these tamponades are amphiphilic (either hydrophilic or lipophilic). The capacity to penetrate the cellular membranes depends on the lipophilic property, and this is directly proportional to the length of the alkylic chain. A minimal increase in the lipophilic properties of some semifluorinated alkanes may lead to their penetration into the cell membranes, causing cellular damage and complete disorganization of the retinal layers and lens structure [30].

However, experimental studies have produced a new biocompatible perfluoroalkane, F4H5 (perfluorobutylpentane). The combination of F4H5 with PDMS 100.000 mPas gave rise to a new HSO, HWS 46-3000. This oil is very viscous (3109 mPas); it did not show a tendency to emulsify in clinical trials, and it is well tolerated. However, its high viscosity limits its use because the removal of this oil is reportedly difficult and time-consuming [42].

### 2.4. Heavy Silicone Oils

#### 2.4.1. Oxane HD

Oxane HD (Bausch and Lomb, Toulouse, France) is a mixture of 5700 mPas PDMS and RMN-3 (perfluorooctyl-5-methyl-hex-2-ene), a mixed fluorinated and hydrocarbonated olefin. The surface tension and interfacial tension of this agent against water are similar to those of perfluorocarbon liquids (41 mN/m), and its specific gravity is only slightly greater than that of water (1.02 g/cm^3^). Its high viscosity (3800 mPas) reduces the risk of early emulsification. The rate of inflammatory reactions related to the use of Oxane HD was reported to be from 3% to 37% of treated patients (Table 1).

The immunogenicity of Oxane HD was investigated in a recent study in which immunohistochemistry was performed on epiretinal membranes formed in redetached retinas under this HSO [80]. Using monoclonal antibodies against retinal pigment epithelium cells, glia, macrophages, and T-lymphocytes, the inflammatory cell population was found to be similar to that obtained with conventional silicone oils; however, several aspects emerged that were attributed to a reaction against a foreign body. CD68-positive macrophages and epithelioid cells containing phagocytosed silicone oil were found in the area adjacent to the fibrocellular component of the membrane.

Another study that investigated intraocular inflammation following endotamponade with Oxane HD showed that 37% of treated patients presented with a severe inflammatory reaction that assumed the characteristics of a granulomatous anterior uveitis [37]. Seven patients in this series developed pigmented endothelial precipitates, flare, and cellularity of the aqueous humor. In contrast to what has been shown in other studies, the uveitic reaction did not regress after the administration of topical corticosteroids and was reversible only after tamponade removal. The immune reaction was attributed to a granulomatous type IV reaction, in which an immune complex of insoluble antigens can cause T-lymphocyte-mediated reaction.

The high percentage of intraocular inflammation in this series was probably due to the intraoperative contact between the Oxane HD and the PFCL. In fact, other authors did not report this phenomenon. Thus, a direct exchange between PFCL and Oxane HD has been contraindicated, and a PFCL-air exchange is recommended before injecting the HSO.

Several case series performing this maneuver did not report uncommon posterior chamber reactions; thereby it was concluded that Oxane HD is well tolerated by the eye for up to 3 months of the endotamponade period [71, 72, 74].

#### 2.4.2. Densiron 68

Densiron 68 (Fluoron, Neu Ulm, Germany) is an admixture of F6H8 (30.5%) and PDMS 5000 mPas (69.5%); thereby, the viscosity was increased to 1387 mPas. This translates into a reduced ability for dispersion and emulsification, consequently reducing irritability to ocular structures [41].

Hence, compared with F6H8 alone, Densiron 68 is associated with significantly less inflammatory side effects [24] (Table 1). A comparison of Densiron 68 with 1000 mPas PDMS demonstrated that Densiron 68 does not have a higher rate of postoperative inflammation in the middle period [87].

Moreover, in cases likely to develop PVR, Densiron 68 was demonstrated to be useful for avoiding repeated surgeries with scleral buckle usage [77, 87]. A common finding was a mild-to-moderate anterior chamber reaction [25, 73, 82]. This inflammatory reaction was sometimes associated with the development of fibrous membranes, the appearance of keratic precipitates, and cataract formation with inflammatory precipitation on the lens. Posterior capsular opacification could be caused by an increased cellular infiltration as a reaction to emulsified tamponade [73, 82].

The percentage of patients who developed significant postoperative inflammation varies greatly in different studies, depending mostly on the tamponade period. The probability of having complications increases if prolonged retention of this agent is required.

A high rate of inflammatory reactions (40.7%) was recorded in a study in which Densiron 68 remained for more than 6 months [79]. In this retrospective study, an inflammatory reaction that was sometimes associated with fibrin exudation or with the appearance of a sterile hypopyon was detected in 11 patients out of 29 affected by complicated inferior retinal detachment.

Due to its low viscosity, Densiron 68 also appears to be correlated with a high rate of dispersion and emulsification in droplets, which in turn precipitates inflammation if a long tamponade period is required [82, 88, 89].

#### 2.4.3. HeavySil (HSIL)

HeavySil (ALCHIMIA srl, Padua, Italy) is made from the combination of high purity 75% silicone oil 5000 cSt (polydimethylsiloxane) and 25% perfluoroalkyloxyoctane (C11H11F13O); it has a density of 1032 and a viscosity of 1500 cSt. Its stability and high affinity for silicone oil are due to the presence of a partially fluorinated ether instead of an alkane.

In a prospective, noncomparative interventional study on 31 consecutive eyes, Romano et al. investigated the anatomic and functional results and complications of this ocular tamponade. They found that HSIL is a safe and effective tamponade agent for the treatment of complicated RD; the main complications were cataract formation (71%), emulsification (19%), sticky oil formation (9.6%), and severe intraocular inflammation (3.2%) (Table 1).

**Figure 1:**
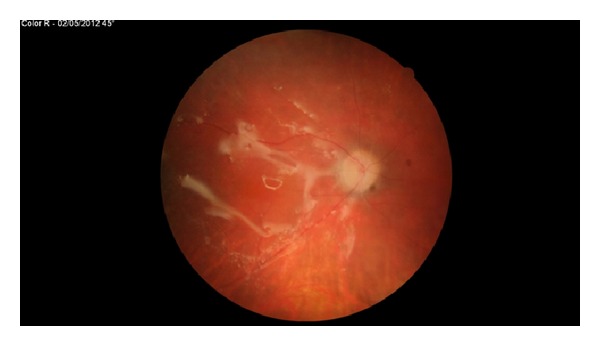
Optic disc swelling in presence of heavySil tamponade.

**Figure 2:**
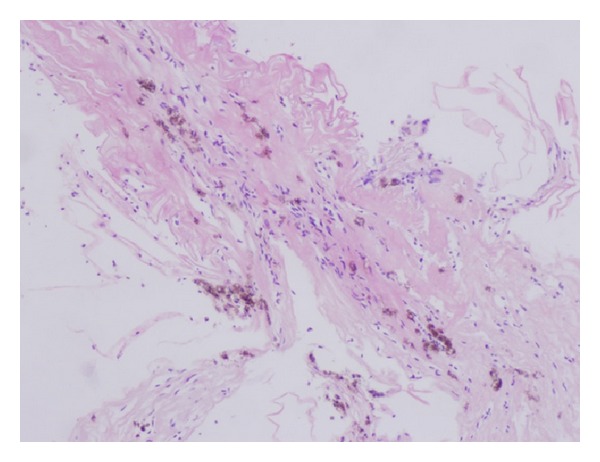
Small specimen of peripheral retinal biopsy showing convoluted basal lamina and retinal microvasculature (arterioles, venules, and intervening capillaries) with prominent reactive endothelium and multiple clusters of pigmented macrophages.

**Figure 3:**
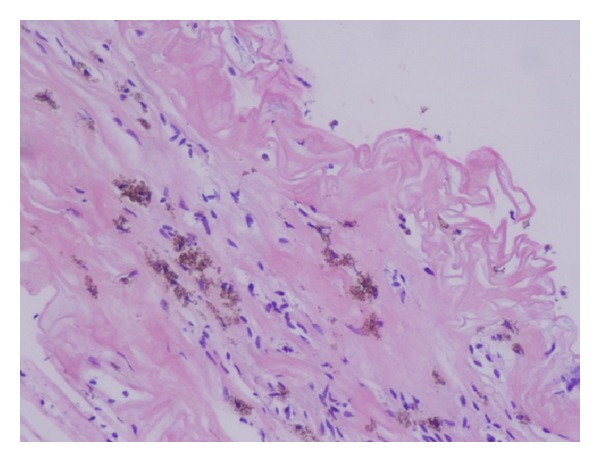
Convoluted basal lamina and retinal microvasculature with reactive endothelium and many pigmented macrophages.

**Figure 4:**
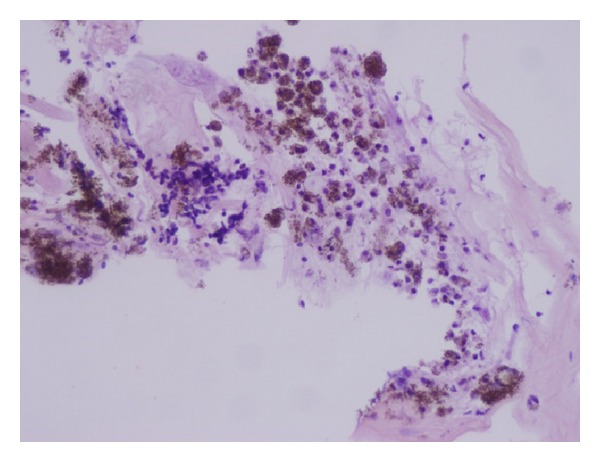
Large aggregates of pigmented macrophages with interdispersed not pigmented histiocytes on the left perivascular lymphoid infiltrate.

One of the coauthors (B. Parolini) reviewed retrospectively 13 eyes of 13 patients with retinal detachment complicated with inferior PVR, treated using HeavySil 1500 as tamponade. All surgeries were performed with standard three-port 20-gauge pars plana vitrectomy. Additional surgical procedures such as membrane peeling and relaxing retinotomy were performed when necessary to allow retinal reattachment. Retinal breaks were treated by endophotocoagulation. In patients with preexisting endotamponade, the silicone oil was removed first. All patients were pseudophakic and underwent already at least one previous vitreoretinal surgery. Three patients were lost at 16-month follow-up. After tamponade with HeavySil, retina appeared to be attached in 9 cases over 10 (90%). Only one patient developed an IOP increase that was successfully treated with topical therapy. Another patient presented with emulsification in anterior chamber. Persistent subretinal fluid was never detected after surgery. Mean best corrected visual acuity was 2.1 ± 0.2 logMar preoperatively and 0.9 ± 0.1 logMar postoperatively. Three cases developed severe retinal inflammation 2 weeks after Heavysil 1500 tamponade. All three patients presented with optic disc swelling and retinal edema with diffuse narrowing of arteries and veins (Figure 1). One patient developed pain and the other two developed significant discomfort. Another case showed retinal inflammation with features resembling herpes retinitis, although virology was negative. In all cases, oil removal was performed within 1 week after the occurrence of retinal inflammation. The appearance of the fundus slightly improved within 2 weeks after oil removal. Silicone oil was analysed in these three cases with cytology and only in one case inflammatory cells were found. During oil-removal surgery, the sticky oil phenomenon appeared in one case. A retinal tissue sample was collected for histology examination; however, the result showed nonspecific signs of inflammation (Figures 2, 3, and 4). In this particular group visual acuity remained very low after surgery even if retinal reattachment was reached in all patients. The cause for final low vision was cystoid edema in one patient and persistent macular hole and retinal thinning in the other two. It is difficult and often impossible to distinguish between problems caused by the tamponade and those that are associated with the underlying complicated retinal disease. In these three cases the timing of acute appearance of inflammation and retinitis was considered significant and differed from other more chronic and subtle signs of silicone related inflammatory reactions. Our case series, with all the limitations due to the retrospective examination of data, shows a significant rate of severe acute retinal inflammation when using HeavySil (70%). Larger prospective clinical trials will be needed in order to define the safety of this new heavy tamponade.

### 2.5. HWS 46-3000 and HWS 45-3000

HWS 46-3000 and HWS 45-3000 are admixtures of 45% silicone oil 100,000 and 55% perfluorobutylhexane (F4H6) and perfluorobutylpentane (F5H6), respectively. Of the three new generation tamponades, HWS 46-3000 is the heaviest and has the greatest viscosity (3.109 mPas). In Rizzo's pilot study of a case series of 32 patients published in 2003, the major side effect detected was the development of early posterior subcapsular cataract (100%); intraocular inflammation and emulsification were not observed (Table 1). Rizzo et al. postulated that the low rate of postsurgical reproliferation and epiretinal membranes formation (9%) was due to adequate contact with the buffering of the retina, reducing the infiltration of the PVR soup. HWS 45-3000 has a density of 1.118 and a viscosity of 2.903 mPas. In 2010, Rizzo et al. did not observe significant emulsification or a significant inflammatory reaction with this agent.

## 3. Conclusions

The treatment of complex retinal detachments using internal tamponade agents produces successful restoration of vision in many cases. However, the recurrence rates for complicated retinal detachment are as high as 20–25%, and this rate increases in the presence of PVR [4–13]. Although vitreoretinal techniques have been improved over the past years, the rate of PVR has not decreased considerably [90].

PDMS or gas exposes the inferior retina (in the orthostatic position) and the posterior pole (in the supine position) to proinflammatory growth factors and cytokines that may generate epiretinal membranes. Compared to PDMS, heavy tamponades theoretically possess the quality to provide better protection to the posterior pole from PVR [29].

With a heavy tamponade, the head movements during common daily postures are expected to displace the liquid meniscus from the upper retina to the posterior capsule of the lens frequently. In contrast, with PDMS, head movements frequently displace the liquid from the inferior retina to the posterior pole, increasing the risk of damaging the macula. Further, when postoperative posturing is more important, such as in cases of posterior breaks, macular hole in highly myopic eyes, or inferior retinectomies, heavy tamponades are advantageous, especially for patients with orthopedic disability or mental retardation and for children [91, 92].

The hypothetical advantage of using a HSO is that the physical separation of the “PVR soup” from the effector cells (retinal pigment epithelial cells, Müller cells, and fibroblasts of the inferior breaks) inhibits or mitigates fibroplasia. The most important presumed advantage for HSO compared to PDMS is a lower redetachment rate after endotamponade removal and a lower rate of macular redetachments.

However, the preliminary results of a recent multicentric randomized trial failed to demonstrate the real superiority of HSO in comparison with standard PDMS in eyes with proliferative PVR of the lower retina [93].

Regarding final acuity, HSO was neither inferior nor superior to PDMS in almost all clinical series. Further, the rate of PVR in HSO-treated patients was not inferior to that registered for PDMS-treated patients; rather, HSO caused a shift of the PVR to the upper retina above the horizontal meridian [93]. To prevent this complication, several authors proposed performing a prophylactic superior laser photocoagulation, while others suggested a shorter endotamponade period with subsequent silicone oil use [25, 73].

The presence of a subtle meniscus of fluid around bubbles with a specific gravity very close to that of water is probably the main reason for the diffusion of growth factors and cytokines from the inferior breaks to the upper retina, which generates epiretinal proliferation. On the other hand, this subtle meniscus of fluid is essential for the correct K^+^ siphoning of the Müller cells and is necessary for avoiding the functional damage due to the excessive drying of the retinal surface that has been reported for heavier agents or for gas [94–96].

The rates of complications, such an inflammatory reaction, macular epiretinal membranes, IOP rise, cataract, and emulsification of HSOs, seemed to be similar to those in patients treated with PDMS in the middle period. This indicates that the intraocular behavior and tolerance of HSOs and PDMS would be similar if they remained in the eye for 3-4 months. This is an important safety result obtained for HSOs in comparison with all of the previously used heavy agents, because none of these agents could be utilized for such a long period without severe complications.

The clear advantages of using HSOs rather than PDMS are shortening of the surgical time, easy handling, and a reduction in the necessity for utilizing external buckles or macular indents. However, when using HSOs, a strict follow-up period is required and the timing of the endotamponade removal should be respected more strictly in comparison with PDMS. Intraocular inflammation is common if it remains for more than 6 months [79].

The real utility of the use of HSOs depends on the correct selection of the patients for treatment. In a number of situations, such as myopic macular holes with or without retinal detachment, myopic foveoschisis, penetrating ocular injuries with retinal detachment, and inferior giant retinal tears, treatment with a heavy substance is easier and should therefore be the first choice.

Moreover, HSOs offer new strategies for treating very complicated cases of retinal detachment caused by a proliferative process, such as alternating the tamponade agent in two different surgeries (i.e., first using an HSO and using PDMS or gas after a few months), or by combining in a single step the HSO with PDMS to reach a tamponade effect on both the upper and lower retina [97].

A mixed bubble of 70% Densiron 68 −30% PDMS has been recently used to obtain a “filling effect,” suggesting that this strategy could minimize the stress produced by the tractional forces originating from eye movements. However, the results of using HSOs in many of these clinical situations have not yet been evaluated in extensive multicentric clinical trials.

In conclusion, the introduction of HSOs represents an improvement in vitreoretinal techniques because the intraocular tolerance of these agents is good for 3-4 months. Even if the goal to prevent PVR formation is not reached and the visual results obtained with HSOs are comparable and not superior to those obtained with the “old PDMS,” these new agents represent a useful new surgical tool. In the same way that the small gauge vitrectomy represents an improvement over the “old” 20-gauge vitrectomy, the HSOs are better than the “old PDMS” in some clinical situations. Although both agents obtain comparable visual results, the new agent gives similar results while the procedures are performed more easily, with appreciable advantages for both the surgeon and the patient.

## Figures and Tables

**Table 1:** Ocular complications after HSOs use in CRT.

Author, year (reference)	Tamponade	*N*	Pathology	Time to removal	Follow-up	Complications	Conclusions
Wolf et al. 2003 [71]	Oxane HD	33	Complicated RRD of inferior quadrants	Within 3 months	12–16 months	Rise in IOP (18%) Pupillary block (6%) Marked AC inflammation (3%) Retinal hemorrhages (6%)	Complications are similar to those reported with conventional silicone oil

Theelen et al. 2004 [37]	Oxane HD	19	Complicated RRD of inferior quadrants	1–4 months	2–4 months after tamponade removal	Keratic precipitates, pigmented clumps, and anterior chamber cellular reaction (37%) Emulsification (11%)	Inflammatory response resembling granulomatous uveitis; it is likely that Oxane HD is an immunogenic agent

Wong et al. 2005 [41]	Densiron 68	42	Complicated RRD of inferior or posterior quadrants	10–16 weeks	>3 months after tamponade removal	Cataract progression (100%) Rise in IOP (8%) Moderate AC inflammation (8%)	We neither observed clinically significant dispersion nor found any inflammation more than we would except from routine vitreoretinal surgery

Rizzo et al. 2006 [72]	Oxane HD	28	Complicated RRD	45–96 days	6 months after tamponade removal	Cataract progression (38%) Rise in IOP (14%) Tamponade in AC (4%) Membrane formation (54%)	Good intraocular tolerance with few minor complications

Sandner and Engelmann 2006 [73]	Densiron 68	48	Complicated RRD	27–4000 days	103 days after tamponade removal	Dispersion (16%) Emulsification (15%) Hypotony (2%) Ocular hypotension (13%) Glaucoma (10%) Cataract progression (50%) Moderate AC inflammation (21%) Sterile hypopyon (4%)	Compared with conventional silicone oil, a temporary inflammation and early emulsification developed more frequently with Densiron 68

Cheung et al. 2007 [74]	Oxane HD	12	RD secondary to myopic macular hole	3-4 months	9–15 months	Transient rise in IOP (42%) Mild oil emulsification and transient peripheral choroidal detachment (8%)	

Rizzo et al. 2007 [42]	HWS 46-3000	32	Complicated RRD of inferior or posterior quadrants	3 months	6 months after tamponade removal	Early posterior subcapsular cataract (100%) Epiretinal membranes (9%) Rise in IOP (0.3%)	No evidence of emulsification and intraocular inflammation

Sandner et al. 2007 [25]	Densiron 68	12	Complicated primary RRD	33–126 days	400 days after tamponade removal	Emulsification (17%) Ocular hypotension (8%) Glaucoma (17%) Cataract progression (100%) Moderate AC inflammation (33%) Suspected intraretinal gliosis (25%)	

Berker et al. 2007 [75]	Oxane HD	21	Complicated primary RRD	3 months	11.5 months	Rise in IOP (14%) Cataract progression and PVR (19%) Dispersion (9.5%) Rubeosis iridis (14%) Vitreous hemorrhage and optic atrophy (5%) Ocular pain and photophobia (100%)	Its complications were acceptable, and mostly due to its physical properties

Boscia et al. 2008 [76]	Oxane HD	10				Posterior capsular opacification (22%) ERM (30%)	

Romano et al. 2008 [77]	Densiron 68	41	Complicated RRD of inferior quadrants	3 months	6 months	AC shallowing (5%) Emulsification (5%) Posterior synechiae (5%) Cataract progression (54%) Rise in IOP (2.4%) PVR (2%)	Analyzing the observed side effects in our series, we found no presence of clinically significant emulsified Densiron 68 or intraocular inflammation

Majid et al. 2008 [78]	Densiron 68	40	Complicated or primary RRD	9–14 weeks	6–12 months after tamponade removal	Emulsification (20%) Fibrinous uveitis (5%) ERM (12%) Cataract progression CMO (8%)	Emulsified Densiron may have contributed to significant intraocular inflammation, ERM formation, and CMO. This has potentially significant implications on the indications for Densiron-68 use.

Auriol et al. 2008 [79]	Densiron 68	27	Complicated primary RRD	14 weeks	6 months	AC inflammation with fibrin accumulation (41%) Sterile hypopyon (1%) PC inflammation with preretinal membranes without traction (0.3%) Hyphema and endothelial corneal dystrophy (1%) Rise in IOP (25%) Emulsification with pseudohypopyon (1%)	Special attention must be paid to unusual adverse effects like inflammatory reactions and fibrin accumulation in the anterior chamber.

Wickham et al. 2010 [80]	Oxane HD	18	Complicated RRD of inferior quadrants	3 months	6 months	Postoperative PVR (28%) Hypotony (17%) Uveitis (11%) Rise in IOP (22.2%) Glaucoma (6%)	Histopathological analysis showed that the structure and associated inflammatory response of membranes were similar to those observed following the use of conventional oils

Meng et al. 2010 [81]	Oxane HD	40	Complicated RRD	Mean 87.9 days	Mean 438.1 days after tamponade removal	Mild-to-moderate AC inflammation (45%) Fibrin accumulation (40%) Temporary pupillary synechiae (15%) Rise in IOP (17.5%) Emulsification and dispersion (22.5%) Cataract progression (77.8%) ERM (30%)	Oxane HD showed an encouraging anatomical and functional success rate and good intraocular tolerance, with a few complications in complicated RD patients

Li et al. 2010 [82]	Densiron 68	21	Complicated primary RRD	1–3 months	15 months	Mild-to-moderate AC inflammation (24%) Severe intraocular inflammatory reaction (10%) Emulsification (24%) and pseudohypopyon (10%) Early posterior capsular cataract (24%) Choroidal detachment/ocular hypotension (5%) Rise in IOP (20%) Pupillary block (5%) Transient corneal edema (5%)	Vitreoretinal surgery with temporary Densiron 68 intraocular tamponade appears to increase anatomical success, while giving rise to minimal complications, in selected cases of complicated RD and PVR

Li et al. 2010 [83]	Densiron 68	27	Complicated RRD of inferior or posterior quadrants	3 months	15 months	Cataract progression (25%) or early posterior capsular opacification (26%) Intraocular inflammation (22%) Corneal edema and rubeosis iridis (7%) Emulsification and dispersion (19%) Rise in IOP (19%) Intraocular hemorrhage (4%) Choroidal detachment/ocular hypotension (11%) Pupillary block (4%) PVR (15%) and ERM (7%)	Postoperative complications did not increase significantly in the vitreoretinal surgery with temporary Densiron 68 intraocular tamponade

Ang et al. 2010 [84]	Oxane HD	18	Complicated RRD of inferior quadrants	Mean 27 weeks	Mean 66 weeks	Posterior capsular opacification (22%) Severe intraocular inflammation (6%) Pseudohypopyon (6%) Emulsification (33%) Rise in IOP (11%) ERM (28%)	The relatively high rate of emulsification and increased risk of intraocular complication when compared to other reported series is less promising and warrants further evaluation

Rizzo et al. 2011 [85]	HWS-45 3000	10	Complicated RRD of inferior quadrants	2 months	6 months after tamponade removal	Tiny droplets in CA (30%) Mild inflammatory reaction (40%)	HWS-45 3000 appears to be a well-tolerated heavy oil suitable for the treatment of complicated inferior retinal detachment

Romano et al. 2013 [86]	HeavySil (HSIL)	31	Complicated RRD of inferior quadrants	1 month	2 months after tamponade removal	Emulsification in AC (19%) Mild/severe intraocular inflammation (9.6%) Cataract formation (71%) Sticky oil formation (9.6%) Posterior synechiae (3.2%)	HeavySil is a safe and effective tamponade agent for the treatment of complicated RD; no major complication in terms of corneal damage, hypotension or hypertension, or postoperative proliferation was observed

Kocak and Koc 2013 [87]	Densiron 68	31	Complicated RRD of inferior quadrants	3 months	6 months after tamponade removal	Rise in IOP (25%) Cataract progression (80%) AC inflammation with fibrin accumulation (3%) PVR (17%) and ERM (6%)	Densiron 68 does not have higher complication rates than conventional silicone oil
